# Polymorphism in a high-entropy alloy

**DOI:** 10.1038/ncomms15687

**Published:** 2017-06-01

**Authors:** Fei Zhang, Yuan Wu, Hongbo Lou, Zhidan Zeng, Vitali B. Prakapenka, Eran Greenberg, Yang Ren, Jinyuan Yan, John S. Okasinski, Xiongjun Liu, Yong Liu, Qiaoshi Zeng, Zhaoping Lu

**Affiliations:** 1 State Key Laboratory for Advanced Metals and Materials, University of Science and Technology Beijing, Beijing 100083, China; 2 Center for High Pressure Science and Technology Advanced Research, Pudong, Shanghai 201203, China; 3 Center for Advanced Radiation Sources, University of Chicago, Chicago, Illinois 60437, USA; 4 X-ray Science Division, Advanced Photon Source, Argonne National Laboratory, 9700 South Cass Avenue, Argonne, Illinois 60439, USA; 5 Advanced Light Source, Lawrence Berkeley National Laboratory, Berkeley, California 94720, USA; 6 Department of Earth and Planetary Sciences, University of California, Santa Cruz, Santa Cruz, California 95064, USA; 7 State Key Laboratory of Powder Metallurgy, Central South University, Changsha, Hunan 410083, China; 8 School of Materials Science and Engineering, Southeast University, Nanjing 211189, China

## Abstract

Polymorphism, which describes the occurrence of different lattice structures in a crystalline material, is a critical phenomenon in materials science and condensed matter physics. Recently, configuration disorder was compositionally engineered into single lattices, leading to the discovery of high-entropy alloys and high-entropy oxides. For these novel entropy-stabilized forms of crystalline matter with extremely high structural stability, is polymorphism still possible? Here by employing *in situ* high-pressure synchrotron radiation X-ray diffraction, we reveal a polymorphic transition from face-centred-cubic (*fcc*) structure to hexagonal-close-packing (*hcp*) structure in the prototype CoCrFeMnNi high-entropy alloy. The transition is irreversible, and our *in situ* high-temperature synchrotron radiation X-ray diffraction experiments at different pressures of the retained *hcp* high-entropy alloy reveal that the *fcc* phase is a stable polymorph at high temperatures, while the *hcp* structure is more thermodynamically favourable at lower temperatures. As pressure is increased, the critical temperature for the *hcp*-to-*fcc* transformation also rises.

## Abstract (web summary)

Whether a polymorphic transition exists in high entropy alloys or not remains unclear since discovery of these alloys more than a decade ago. Here authors report an irreversible polymorphic transition from *fcc* to *hcp* in the prototype FeCoCrMnNi high entropy alloy and provide evidence for *fcc* phase being more stable than hcp phase only at high temperatures.

Polymorphism, which refers to the ability of a solid material to exist in more than one crystal structure, is widely observed in many materials including polymers^1^, minerals^2^,^3^,^4^ and metals^5^,^6^,^7^,^8^. The polymorphic transition is a well-known yet critical phenomenon in nature and surprisingly, polymorphs with identical compositions can have different physical, chemical and mechanical properties due to different structural characteristics, which have attracted considerable attention over the last few decades^9^,^10^,^11^. Pressure and temperature are usually controlling parameters for inducing these transitions by altering the atomic and/or electronic structures and creating novel materials. For example, carbon can take on graphite and diamond under different pressures and temperatures.

Recently, a new alloy design concept based on entropic contributions to the thermodynamic landscape, rather than cohesive energy, was proposed^12^,^13^, which was subsequently extended to non-metallic systems such as multicomponent oxides^14^. This strategy was explored by deliberately incorporating five or more elemental species into a single lattice with random site occupancy. Due to the engineered configuration disorder, these entropy-stabilized solid solution alloys, that is, high-entropy alloys (HEAs), usually show a single-phase structure with high symmetry and extremely attractive properties^15^,^16^,^17^,^18^,^19^,^20^,^21^. Among various reported HEAs in literature, the equiatomic CoCrFeMnNi alloy (also termed as Cantor's alloy)^12^,^13^ is a prototype *fcc* (face-centred-cubic) HEA, which possesses ultrahigh fracture toughness and large ductility, even at cryogenic temperatures^19^,^22^,^23^. It is generally believed that HEA lattices are severely distorted and atomic diffusion is extremely sluggish due to the chemical complexity and packing disorder^24^,^25^. Consequently, HEAs have exceptionally high microstructural stability^26^,^27^. Many studies also demonstrate that the CoCrFeMnNi HEA can maintain its *fcc* structure over a large temperature range from cryogenic temperatures up to the melting temperature without any polymorphic phase transition^19^,^22^,^28^. Considering the extensive polymorphism in the typical constituent elements of HEAs, such as Fe, Co and Mn^29^, an intriguing question is naturally raised: when metallic alloys are made in the entropy-stabilized form of crystalline matter with high structural stability, is polymorphism still possible?

More recently, long-term annealing experiments at intermediate temperatures revealed that different crystalline phases could precipitate out of the CoCrFeMnNi HEA *fcc* matrix^27^,^30^,^31^. Moreover, finite-temperature *ab initio* simulation results suggest that the free energy of the *hcp* (hexagonal-close-packing) crystallographic lattice in the CoCrFeMnNi HEA may be lowest at room temperature, compared with that of its bcc (body-centerd-cubic) and *fcc* counterparts^32^,^33^. All these results cast doubt on the high structural stability of HEA lattices, which is obviously a critical and vital problem for our understanding and applications of these newly developed metallic materials and multicomponent oxides. However, until now the predicted *hcp* phase of the CoCrFeMnNi HEA has not been observed in any experiments and the question of viable polymorphism remains unanswered.

In this work, we employed an *in situ* high-pressure synchrotron radiation X-ray diffraction (XRD) technique and discovered a polymorphic transition from *fcc* to *hcp* in the prototype CoCrFeMnNi HEA under hydrostatic compression at room temperature. The phase transition is sluggish, it starts at ∼22 GPa and almost completes at ∼41 GPa, and the *hcp* phase could be retained when the pressure was totally released, which is the first synthesis of the CoCrFeMnNi HEA with an *hcp* structure at ambient conditions. Our *in situ* laser/resistive heating XRD experiments at different pressures show an inverse transition from *hcp* back to *fcc* at elevated temperatures and reveal that the *fcc* phase is stable at high temperatures while the *hcp* structure is more thermodynamically favourable at relatively lower temperatures. Our findings indicate that this *fcc* HEA is a thermodynamically metastable polymorph obtained by kinetic constraints and the thermodynamically favourable *hcp* structure is relatively difficult to attain.

## Results

### Microstructure and compositional analysis

The CoCrFeMnNi HEA sample was synthesized using a gas-atomization technique^34^. The gas-atomized powders are spherical particles with a typical size ranging from a few microns to tens of microns, which are appropriate for high-pressure experiments (Supplementary Fig. 1). These particles are polycrystalline with a grain size ranging from submicron to a few microns (the inset of Supplementary Fig. 1). The chemical and energy dispersive X-ray spectroscopy analyses confirm that the composition of the as-atomized CoCrFeMnNi HEA is close to the nominal composition, and there are no compositional changes after the high-pressure XRD tests (Supplementary Fig. 2 and Supplementary Table 1).

### *In situ* high-pressure synchrotron XRD at room temperature

**Figure 1:**
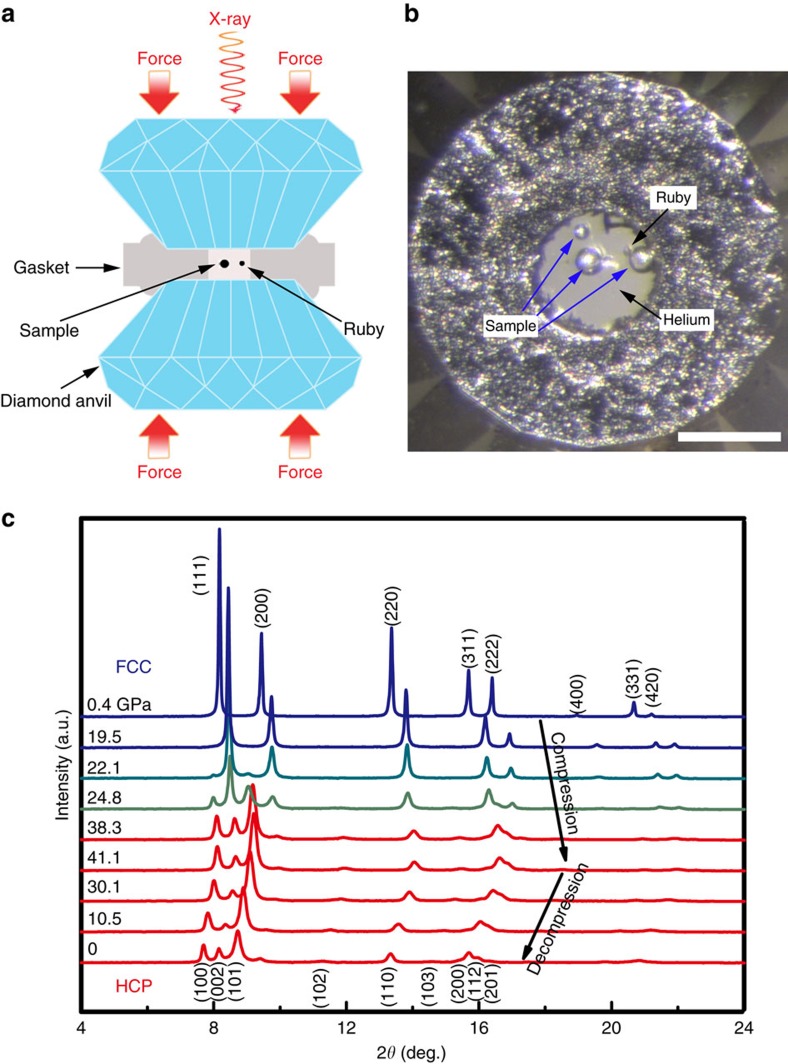
Experimental set-up and the *in situ* high-pressure XRD patterns of the CoCrFeMnNi HEA in a DAC during compression and decompression at room temperature. (**a**,**b**) A schematic illustration of the DAC used to generate high-pressure on tiny samples for the *in situ* high-pressure experiment. (**c**) XRD patterns as a function of pressure obtained during compression and decompression with a X-ray wavelength *λ*=0.2952 Å. The initial phase is indexed to an *fcc* lattice, whereas the new phase synthesized above 22 GPa is well indexed to an *hcp* lattice. Intensity mismatch of the standard *fcc* and *hcp* structures is caused by the relatively large grains in the samples. The scale bar in **b** represents 100 μm.

**Figure 2:**
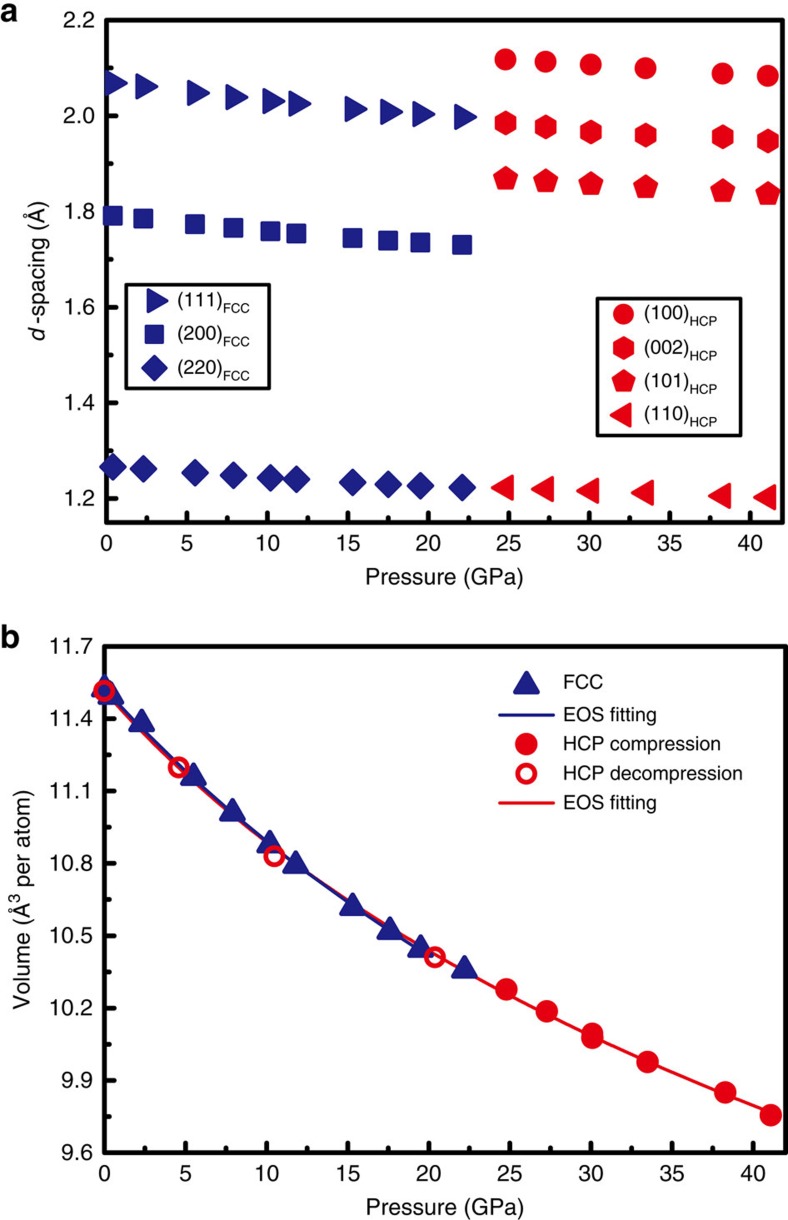
Pressure dependence of the CoCrFeMnNi HEA *d*-spacings and volume. (**a**) The pressure dependence of the *d*-spacings associated with the major Bragg peaks of *fcc* (shown in blue symbols) and *hcp* (shown in red symbols), respectively. The error bars are smaller than the symbol sizes. (**b**) The average volume per atom for both the *fcc* (blue triangles) and *hcp* phases (red solid circles for compression and red open circles for decompression) are calculated as a function of pressure. The volume data can be well fitted using the third-order Birch-Murnaghan EOS for both *fcc* (blue solid line) and *hcp* (red solid line). The EOS of *fcc* and *hcp* almost coincides with each other, showing no obvious volume difference at any given pressure.

Hydrostatic pressure up to ∼41 GPa was applied to the tiny CoCrFeMnNi HEA particles using a diamond anvil cell (DAC). The experimental set-up of the DAC and the sample loading image are shown in Fig. 1a,b, respectively. More details can be found in Methods. The structural evolution of the CoCrFeMnNi HEA during compression was monitored by high-brightness synchrotron radiation XRD through two transparent diamond anvils of the DAC. The XRD patterns collected from both the compression and decompression processes are shown in Fig. 1c. The initial HEA sample has an *fcc* single-phase structure, which remains stable up to 19.5 GPa. All diffraction reflections shift towards higher 2*θ* values, which is an expected result of the pressure-induced volume decrease. Once the pressure approaches 22.1 GPa, extra diffraction peaks beside the (111) peak of *fcc* appeared (indicated by red triangles in Fig. 1c), suggesting the occurrence of a phase transition. With further increasing pressure, the intensity of the *fcc* peaks was obviously lowered, while that of the new diffraction peaks increased appreciably. The transition was sluggish, the *fcc* and new phase coexisted over large pressure range. At the maximum pressure of ∼41 GPa, the diffraction reflections mainly corresponded to the new phase while only minute residual *fcc* phase remained. During decompression, surprisingly the phase transition was irreversible. The newly formed phase was completely retained down to ambient pressure. All the new phase peaks were well indexed as crystallographic reflections of an *hcp* lattice (space group *P6_3_/mmc*), which revealed an *fcc*-to-*hcp* transition under high-pressure in the prototype CoCrFeMnNi HEA (Table 1 and Supplementary Fig. 3). Detailed information about their structure, such as the interplanar *d*-spacing (Fig. 2a) and unit cell volume (Fig. 2b) as a function of the applied pressure, was obtained by fitting the measured compression and decompression XRD patterns using the Le Bail method in the standard GSAS package^35^. The *c*/*a* ratio of the *hcp* phase remained almost constant at 1.62±0.01 during both compression and decompression. These two sets of volume data in Fig. 2b can be well fitted by a third-order Birch-Murnaghan isothermal equation of state (EOS)^36^, with the isothermal bulk modulus *B*_0_=153.9±3.0 GPa and its pressure derivative 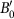
=4.9±0.7 for the *fcc* phase and *B*_0_=150.2±4.6 GPa and 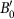
=6.2±0.5 for the *hcp* phase. The bulk moduli obtained in this work are consistent with previous experimental results^28^ on the *fcc* phase and simulation results^33^,^37^ on both the *fcc* and *hcp* phases. The two phases exhibit no obvious difference in both volume and bulk modulus. It should be noted that the grain size of the sample is still not small enough to obtain smooth diffraction rings, as compared with the tiny X-ray beam size (2.5 × 4 μm^2^). Therefore, the relative intensity of the different peaks is not statistically reliable for the Rietveld refinement to accurately derive the volume fractions of the *fcc* and *hcp* phases during the transition. Alternatively, by assuming the phase fraction is proportional to the area of corresponding diffraction peaks, such as the *fcc*-(200) and *hcp*-(101) peak areas in Fig. 1c, the volume fraction of *hcp* as a function of pressure during both compression and decompression can be roughly estimated (Supplementary Fig. 4). The results in Supplementary Fig. 4 confirm that the transition is sluggish over a wide pressure range and irreversible with an almost constant volume fraction of *hcp* during the decompression.

### *In situ* laser/resistive heating synchrotron radiation XRD

**Figure 3:**
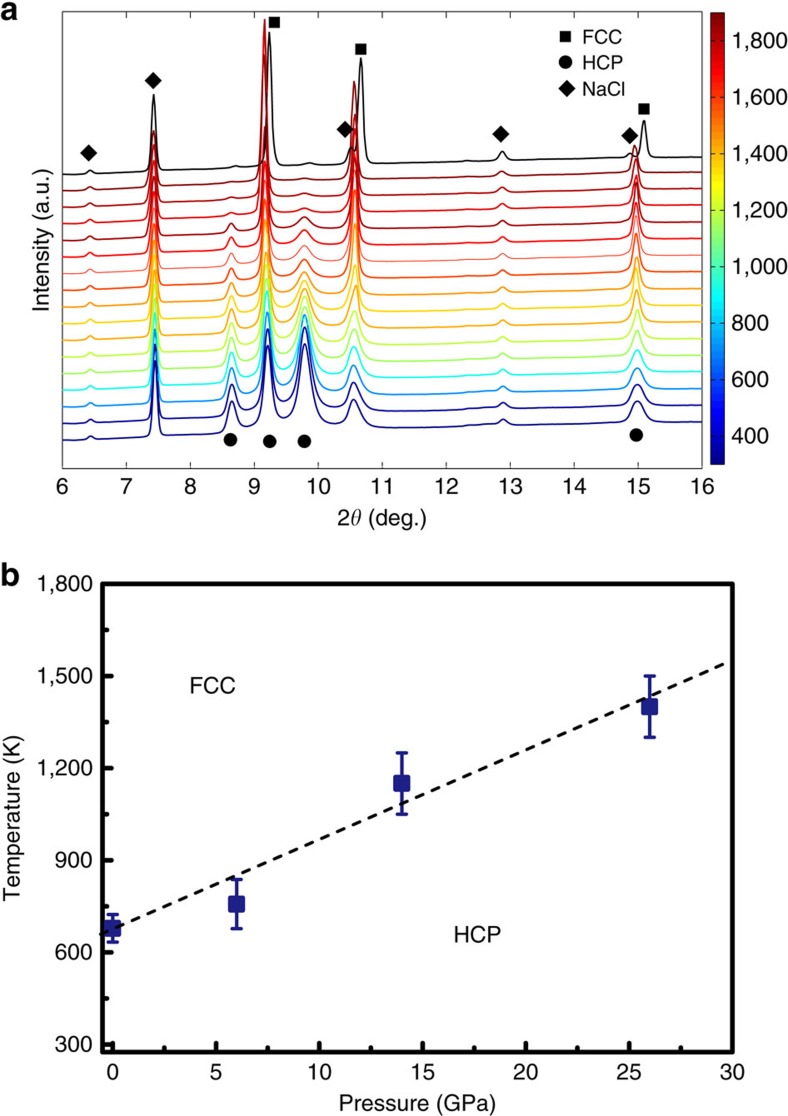
*In situ* laser heating XRD of the CoCrFeMnNi HEA at different pressures. (**a**) The XRD patterns as a function of temperature from room temperature to 1,850 K with an X-ray wavelength of ∼0.3220 Å. The intensity of the major *hcp* peaks is almost constant below 1,400 K, but starts to gradually decrease during continuous heating above ∼1,400 K, indicating the critical transition temperature occurs at ∼1,400 K. (**b**) The temperature and pressure metastability boundary (not the equilibrium phase boundary) of the *hcp* CoCrFeMnNi HEA. The critical transition temperature is determined as the first appearance temperature of the *fcc* phase from the *hcp* phase (synthesis curve of the *fcc* phase) during heating at different pressures and it increases with pressure. The error bars of temperatures were estimated by the fitting errors of the blackbody radiation spectra and the temperature difference between two sides of the sample.

The irreversibility of the phase transition questions the relative stability of the *fcc* and *hcp* phases. To clarify this, *in situ* heating XRD studies were carried out at different pressures on the synthesized *hcp* phase. At ambient pressure, the *hcp* sample was sealed in a resistive heating DAC using silicone oil to prevent oxidation of the tiny sample. The *hcp* phase remained stable up to 633 K at ambient pressure, and almost completely transformed back to *fcc* when heated up to 723 K (Supplementary Fig. 5). Once the pressure was increased, the critical transition temperature significantly increased, which is beyond the limit of the resistive heating DAC. Therefore, a double-sided Nd:YLF laser heating system coupled with a DAC was employed to heat the *hcp* phase at higher pressures of 6, 14 and 26 GPa. As an example, Fig. 3a presents the *in situ* laser heating XRD patterns collected at 26 GPa. With continuous heating above ∼1,400 K at 26 GPa, the *fcc* phase started to gradually grow at the expense of the *hcp* phase. Therefore, the temperature of ∼1,400 K was determined to be the critical temperature (*fcc*-in) for the phase transition. Based on four critical transition temperatures determined at different pressures, a boundary is proposed in Fig. 3b.

## Discussion

The structural stability of the prototype CoCrFeMnNi HEA has been extensively studied in a wide temperature range from 55 K to its melting temperature of ∼1,600 K at ambient pressure^19^,^31^,^37^,^38^ but to our knowledge, no polymorphic transition has been observed. Conversely, high-pressure studies remain a virgin area in HEAs. In this work, using *in situ* high-pressure XRD techniques, we observed the first polymorphic transition from *fcc* to *hcp* in the CoCrFeMnNi HEA, as shown in Fig. 1c. Therefore, some critical questions are how and why can an *hcp* HEA form at high-pressure and can it be maintained at ambient conditions? According to the *d*-spacing of the main reflections of both *fcc* and *hcp* (that is, (111) and (220) of *fcc*, and (100), (002), (101) and (110) of *hcp*, as a function of pressure shown in Fig. 2a), it is clear that the *hcp*-(002) and *hcp*-(110) planes inherit the *fcc*-(111) and *fcc*-(220) planes, respectively. In addition, Fig. 2b shows no obvious volume collapse during the transition; both the *fcc* and *hcp* phases seem to have almost the same volume per atom and bulk modulus. This is similar to many other *fcc* to *hcp* martensitic transitions, such as in cobalt^39^ or xenon^40^, which also show weak first-order features.

It is well known that the *fcc* and *hcp* lattices only differ in the stacking sequence of the close packing layers, for example, the *fcc* and *hcp* crystal lattices follows the pattern of ABCABCABC…along the *fcc* [111] direction and ABABAB…along the *hcp* [001] direction, respectively. The *hcp* structure can be readily obtained locally from a *fcc* structure by introducing stacking faults. And it can be easily realized in the current HEA because recent experiment^41^ and simulations^37^,^41^ have confirmed that it indeed has a low staking fault energy of ∼20 mJ m^−2^. However, the detailed mechanism of this transition could be much complex since the stacking faults, dislocations, and twining events may be all involved, and the magnetic states and volume (high pressure tuning) of the HEA alloy could also affect the transition behaviour^32^,^33^,^42^,^43^.

As shown in Fig. 3, the results of the *in situ* heating XRD experiments at different pressures explicitly demonstrate that the *hcp* phase of the CoCrFeMnNi HEA is relatively stable at low temperatures and high pressures, while the *fcc* phase is actually stable at higher temperatures and lower pressures, showing a boundary in between them with a positive slope. Using *ab initio* calculations, Ma *et al*.^32^ actually revealed that thermodynamically, the CoCrFeMnNi HEA prefers the *hcp* structure below 340 K at ambient pressure and paramagnetic state while the *fcc* phase dominates at higher temperatures, which is qualitatively consistent with our experimental observations. It should be noted that in our experiment the phase transitions were sluggish and irreversible, suggesting high energy barriers between the *hcp* and the *fcc* phases, therefore overshooting often exists. It is difficult to determine the real equilibrium phase boundary (that is, the Clapeyron coexistence curve) experimentally. The curve in Fig. 3b is actually a synthesis curve showing the first appearance of the *fcc* phase (*fcc*-in) from the *hcp* phase. During cooling, the *fcc* phase was fully recovered to room temperature showing a large metastability range. The real equilibrium phase boundary between *fcc* and *hcp* may be much lower than 633 K observed in experiments. According to Ostwald's Rule^44^, high-temperature polymorphs often form first during cooling/crystallization. In other words, with conventional preparation techniques such as melt casting and atomization, the CoCrFeMnNi HEA always forms the *fcc* structure first, and when cooled down to room temperature, the transition from *fcc* to *hcp* likely depends on the entire complex *γ*-surface and could be kinetically hindered^32^,^42^,^45^. However, nano-twinning has been observed in the CoCrFeMnNi HEA at cryogenic temperatures (77 K) (ref. 19). This observation indicates that the stacking fault energy may diminish with decreasing temperature, which could facilitate the sliding needed for a displacive phase transition from *fcc* to *hcp*^32^. Recent theoretical work studied the stacking fault energy as a function of temperature in the CoCrFeMnNi HEA and confirmed this speculation^33^,^37^. Compared with temperature, pressure is a more powerful parameter in terms of decreasing the volume, tuning packing and energy state of a sample. The polymorphic transition from *fcc* to *hcp* unveiled in this work demonstrates that high-pressure can help overcome the kinetic barrier between *hcp* and *fcc* HEAs. Since the *hcp* CoCrFeMnNi HEA is more thermodynamically stable than the *fcc* polymorph at lower temperatures, the *hcp* phase can be retained after pressure release, and this *hcp* phase may have excellent mechanical properties at cryogenic temperatures.

The polymorphic transition discovered in this work is by no means limited to this specific CoCrFeMnNi HEA, and we expect that this behaviour could be general in various HEAs at certain pressure and temperature conditions. Therefore, our results contribute to understanding of thermodynamics and kinetics in HEA systems. Moreover, the relative stability of the *fcc* and *hcp* phases of hard spheres is a long-standing problem in statistical physics. Since the *fcc* and *hcp* have identical packing densities and close free energy, the transition between *fcc* and *hcp* is believed to be entropy dominated. Therefore, the *fcc*-*hcp* transition in HEAs with extremely high entropy could provide ideal model systems for understanding fundamental questions about *fcc* and *hcp* packing^46^. Furthermore, we noticed that both the *fcc* and *hcp* can coexist over a wide pressure range (∼22 to ∼41 GPa), which could be attributed to the very similar free energy of the *fcc* and *hcp* phases and/or the large energy barrier between them. Decompression from different maximum pressures between ∼22 and ∼41 GPa in our repeated experiments successfully obtains *fcc*/*hcp* composites with tunable fractions after pressure release. As recently reported, the *fcc-hcp* dual-phase Fe_50_Mn_30_Co_10_Cr_10_ composites, which undergo a strain-induced martensitic transformation of the initial *fcc* phase, exhibits outstanding mechanical properties that are much better than its monolithic counterpart^15^. However, no such dual phase composite has ever been observed in an equiatomic multicomponent HEA system with maximization entropy. According to the stability curve established in this work, the transition pressure could be largely decreased at cryogenic temperatures. Therefore, our results suggest new avenues for tailoring HEAs properties in their near infinite compositional space for novel applications via polymorphic transition-induced HEA composites.

## Methods

### Sample preparation

The polycrystalline CoCrFeMnNi HEA in our work was synthesized by a previously reported gas-atomization method^34^. Alloy ingots with a nominal composition of Co_20_Cr_20_Fe_20_Mn_20_Ni_20_ were melted in an induction-heated vacuum furnace. The melt was then injected through a ceramic tube into the atomization chamber filled with high purity Ar gas. The atomization pressure was set at 4 MPa. The melt droplets cooled down and solidified into powder. The powder was then collected and sieved.

### Microstructure and chemistry characterization

The composition of the gas-atomized powder was analysed with a titration method, and the oxygen content of the powder was determined by a fusion method on a Leco O/N analyser. The microstructure of the as-atomized specimens was characterized using a Zeiss Supra 55 scanning electron microscope operated at 15 kV with an energy dispersive spectrometer.

### *In situ* high-pressure synchrotron XRD at room temperature

*In situ* high-pressure angle-dispersive XRD experiments were mainly performed at the beamline 13-ID-D, Advanced photon source (APS), Argonne National Laboratory (ANL). A monochromatic X-ray beam with a wavelength of 0.2952 Å was focused by a Kirkpatrick-Baez (KB) mirror system down to approximately 2.5 × 4 μm^2^, in a rectangular shape at the rotation centre of the sample stage. The detector position and orientation were calibrated using the LaB_6_ standard. High-pressure was generated using a symmetric DAC with a 400 μm diameter culet. The gasket was T301 stainless steel that was pre-indented down to a thickness of ∼42 μm. A 180 μm hole was drilled as the sample chamber by a laser drilling system at the centre of the gasket indention. A few polycrystalline CoCrFeMnNi balls with a diameter of approximately 20 μm were selected and loaded into the DAC, along with a tiny ruby ball beside the samples to calibrate pressure. Helium was loaded into the DAC at sector 13, APS, ANL, as the hydrostatic pressure-transmitting medium. Figure 1b shows the image of the sample with helium loaded at 0.4 GPa. The pressure fluctuation estimated before and after each exposure was found to be less than 0.2 GPa. The background scattering was collected at each pressure by shining the X-ray beam on the empty area inside the sample chamber, which only passed through helium and two diamond anvils. Two-dimensional (2D) diffraction images were collected with a Mar165 charge-coupled device detector (pixel size: 79 × 79 μm^2^) and the exposure time was set at 2 s. One-dimensional (1D) XRD patterns were obtained by integrating the 2D images along the azimuth angle from 0° to 360° with Dioptas software^47^. The resulting diffraction patterns were refined via Le Bail refinement using the GSAS package^35^.

### *In situ* resistive/laser heating XRD at different pressures

To check the structural stability of the CoCrFeMnNi HEA *hcp* phase, *in situ* heating XRD experiments were conducted. Resistive heating up to 723 K in a DAC was performed on the high-pressure recovered *hcp* HEA sample at the beamline 12.2.2 of the Advanced Light Source, Lawrence Berkeley National Laboratory. The *hcp*-structured CoCrFeMnNi HEA for the heating experiment was synthesized by compressing the original *fcc*-structured CoCrFeMnNi HEA to 35 GPa and then decompressing it to ambient pressure in a DAC at room temperature. The wavelength of the X-ray was ∼0.4959 Å, and the beam size was 20 × 20 μm^2^. 2D XRD images were collected using a Mar345 image plate. To achieve higher temperatures and study the stability the *hcp* phase at higher pressures, a complementary online laser heating system coupled with DACs at the beamline 13-ID-D, APS, ANL was employed to perform double-sided laser heating on the *hcp* phased sample^48^. The wavelength of the X-ray was ∼0.3220 Å. NaCl was used as the pressure-transmitting medium, pressure standard and thermal insulator layers. Three pressure points of 6, 14 and 26 GPa were tested. Temperatures were determined by fitting the blackbody radiation spectra of the heated sample on both sides to the Planck radiation function.

### Data availability

The data that support the findings of this study are available from the corresponding authors upon request.

## Additional information

**How to cite this article:** Zhang, F. *et al*. Polymorphism in a high-entropy alloy. *Nat. Commun.* **8**, 15687 doi: 10.1038/ncomms15687 (2017).

**Publisher's note**: Springer Nature remains neutral with regard to jurisdictional claims in published maps and institutional affiliations.

## Supplementary Material

Supplementary Information Supplementary Figures and Supplementary Table

## Figures and Tables

**Table 1:** Unit-cell parameters and *d*-spacings of the different (*hkl*) planes of both the *fcc* and *hcp* CoCrFeMnNi HEAs at different pressures.

**(*hkl*)**	***fcc***				**(*hkl*)**	***hcp***			
	***d*_obs_, (Å)**	***d*_cal_, (Å)**	***d*_obs_, (Å)**	***d*_cal_, (Å)**		***d*_obs_, (Å)**	***d*_cal_, (Å)**	***d*_obs_, (Å)**	***d*_cal_, (Å)**
111	2.071	2.073	2.005	2.003	100	2.081	2.083	2.200	2.196
200	1.794	1.795	1.737	1.735	002	1.954	1.947	2.075	2.069
220	1.268	1.269	1.228	1.226	101	1.838	1.837	1.941	1.939
311	1.082	1.083	1.048	1.046	110	1.202	1.202	1.271	1.267
*P*, GPa	0.4		19.5			41.1		0.0	
*a*, *c*, Å	3.582(1)		3.470(1)			*a*=2.405 (1)		*a*=2.535(2)	
						*c*=3.895(1)		*c*=4.138(1)	
*V*, Å^3^	11.493(1)		10.444(1)			9.7551(1)		11.5145(2)	

The subscript ‘obs' represents experimentally observed values and ‘cal' represents values from the Le Bail refinement.
