# Supplementary material for: Polymorphism in a high-entropy alloy
Source: Nat Commun. 2017 Jun 1;8:15687. doi: 10.1038/ncomms15687 (PMC5461481; doi:10.1038/ncomms15687)
Supplement: Supplementary Information — Supplementary Figures and Supplementary Table [file ncomms15687-s1.pdf]

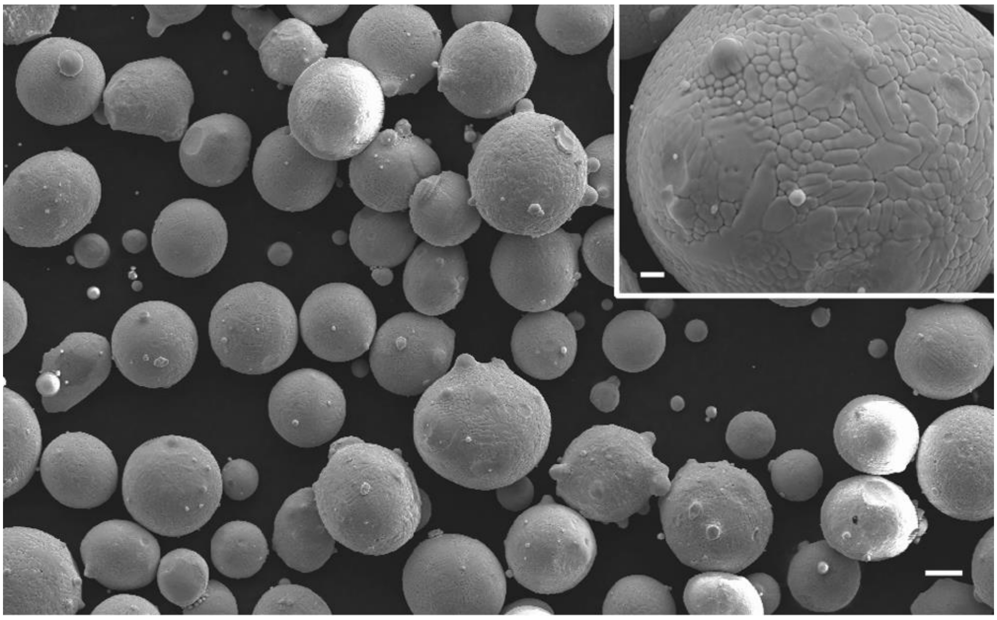

Supplementary Figure 1. Microstructure of the as-atomized CoCrFeMnNi HEA powder. The powder consists of spherical particles with sizes ranging from a few microns to tens of microns. The inset is a blowup of an individual particle, indicating that it is polycrystalline with a grain size ranging from submicron to a few microns. The scale bars represent 10 μm and 2 μm in the inset.

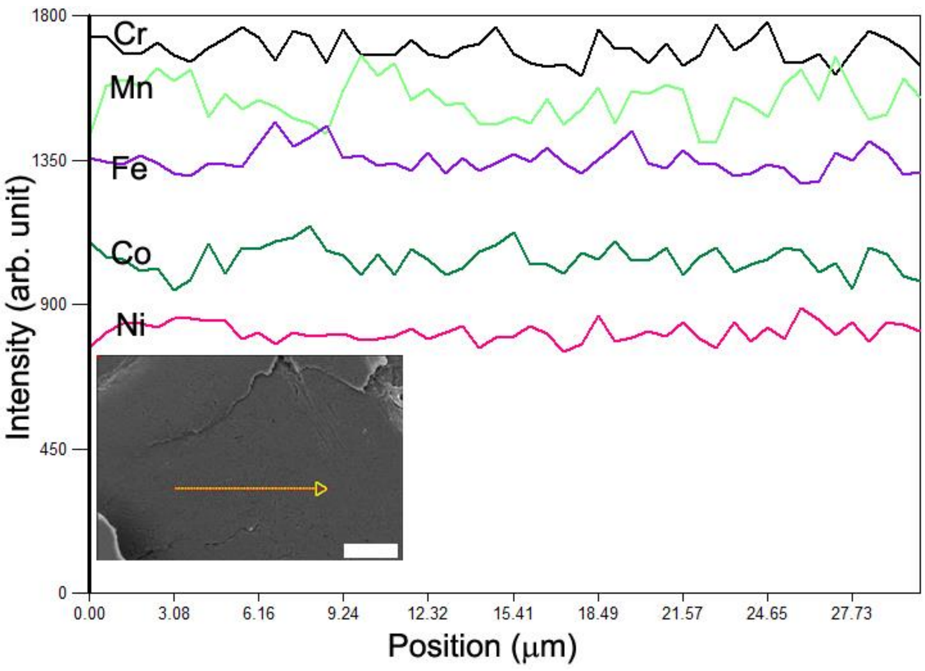

Supplementary Figure 2. Compositional analysis of *hcp* CoCrFeMnNi HEA retained after a high-pressure test. The EDS results, obtained by scanning along the line indicated in the inset, show a homogenous spatial distribution of all five constituent elements, indicating no compositional change after the test. The inset shows the location of the line scanning on the sample. The scale bar represents 10 μm in the inset.

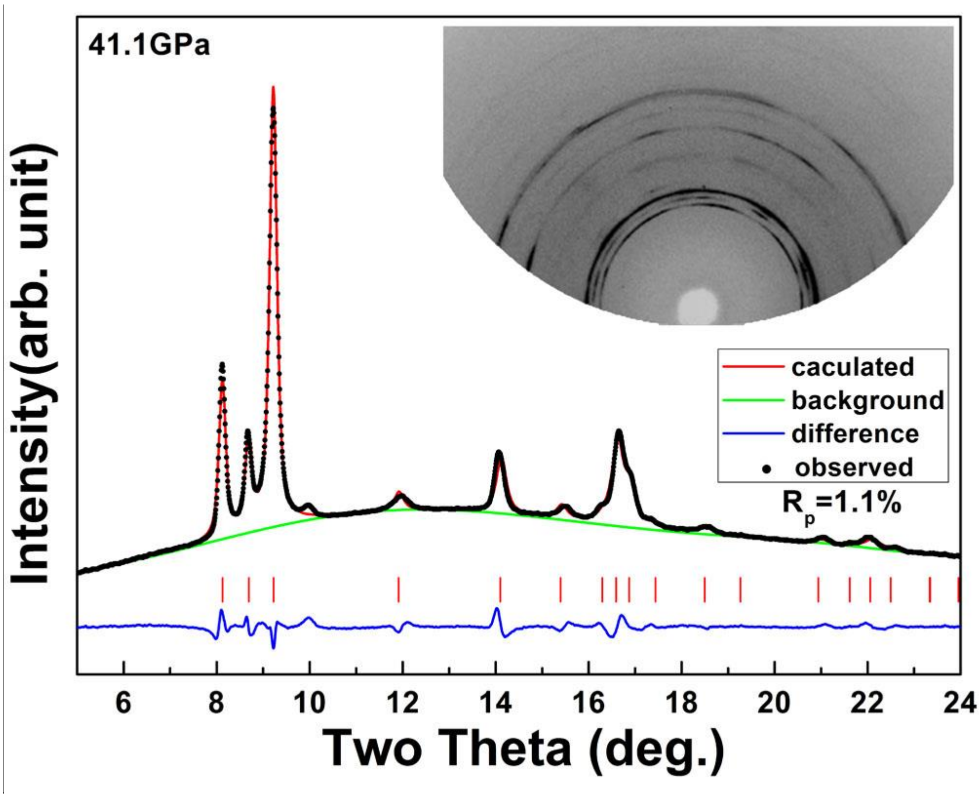

Supplementary Figure 3. **Representative Le Bail refinement of CoCrFeMnNi HEA at 41.1 GPa.** The experimental (black dots) and calculated (red curve) data match very well. The relative large R_p value is mainly caused by the residual minute *fcc* phase. The red vertical lines show the positions of the allowed Bragg reflections for the *hcp* lattice. The inset shows the two-dimensional diffraction image of this particular sample. The intensity along each diffraction ring is not smoothly distributed because of the relatively large grain size in the initial samples. As a result, the intensity of the diffraction peaks is unreliable for Rietveld refinement. The X-ray wavelength is ~0.2952 Å.

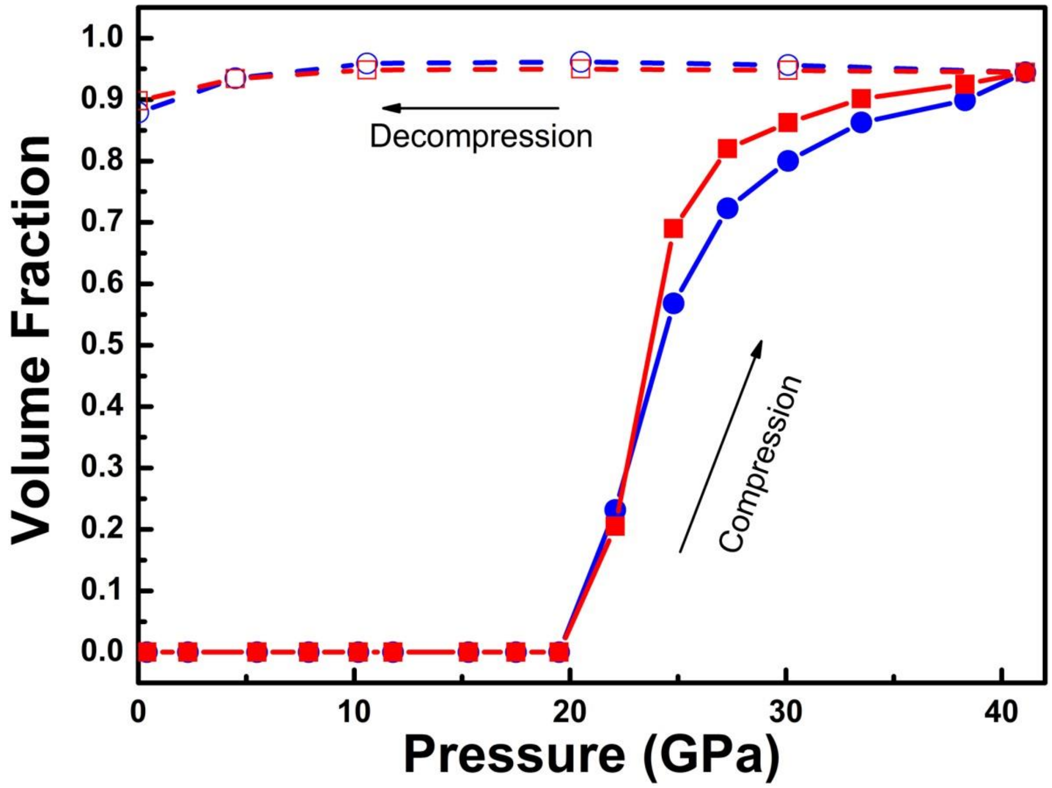

Supplementary Figure 4. **Volume fraction of *hcp* phase as a function of pressure during compression (solid symbols) and decompression (open symbols)**. Since the phase fraction is proportional to the area of corresponding diffraction peaks, the volume fractions the *hcp* phase were calculated based on the peak area changes of the *fcc*-(200) (shown in blue circles) and *hcp*-(101) peaks (shown in red squares) in Fig. 1c, which yield consistent results. It can be found that the *fcc* to *hcp* transition is sluggish over a wide pressure range from ~ 20 GPa to ~ 41 GPa, and minute residual *fcc* phase (~ 6%) was not fully converted into *hcp* phase even up to 41.1 GPa at room temperature. During compression, the transition is irreversible with almost constant volume fraction of *hcp*. Since the sample has relative large grains, the diffraction peak intensity can be also affected by the sample position where the x-ray beam shined, the sample position remains stable during almost the entire experiment, and that's why the consistent and reasonable volume fractions were obtained based on either the *fcc*-(200) or *hcp*-(101) peak areas. The slight decrease of the volume fraction (~ 4%) at 0 GPa during decompression may be caused by the sample movement during the sudden helium evaporation when the pressure approaches 0 GPa.

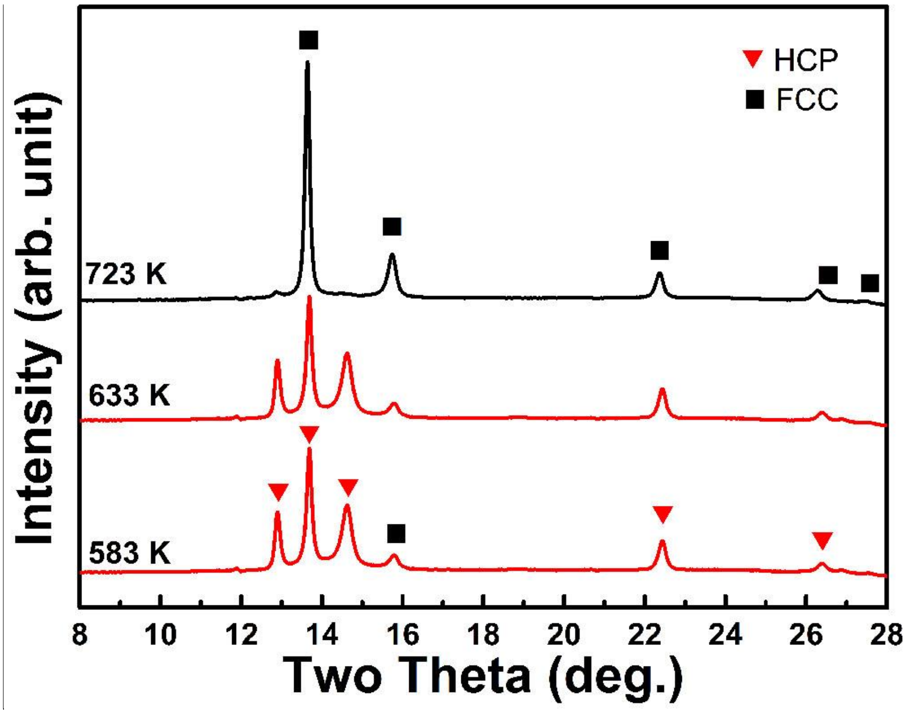

Supplementary Figure 5. **XRD patterns of CoCrFeMnNi HEA in an external resistive heating DAC at ambient pressure.** The diffraction peaks of the *fcc* and *hcp* phases are indicated by black squares and red triangles, respectively. A thermally driven transition from *hcp* to *fcc* occurs between 633 K and 723 K.

Supplementary Table 1. Compositions of the initial *fcc* sample and the high-pressure synthesized *hcp* sample. They are almost identical within experimental uncertainty.

Elements (at.%)	Co	Cr	Fe	Mn	Ni
Before HP	18.4	19.1	19.5	20.9	22.1
After HP	19.4	19.7	19.1	20.6	21.2
